# Supplementary material for: Resolving the full spectrum of human genome variation using Linked-Reads
Source: Genome Res. 2019 Apr;29(4):635–45. doi: 10.1101/gr.234443.118 (PMC6442396; doi:10.1101/gr.234443.118)
Supplement: Supplemental Material [file supp_gr.234443.118_Supplemental_longranger-2.2.zip › longranger-2.2.tar.bz2!/lr2.2-public/lib/stan/cmdstan/stan/doc/api/html/agrad_2rev_2matrix_2tcrossprod_8hpp_source.html]

Stan: src/stan/agrad/rev/matrix/tcrossprod.hpp Source File


|  |  |
| --- | --- |
|  | Stan  2.5.0  probability, sampling & optimization |


- Main Page
- Related Pages
- Namespaces
- Classes
- Files

- File List
- File Members

All Classes Namespaces Files Functions Variables Typedefs Enumerations Enumerator Friends Macros Pages

- src
- stan
- agrad
- rev
- matrix

tcrossprod.hpp

Go to the documentation of this file.

1 #ifndef STAN\_\_AGRAD\_\_REV\_\_MATRIX\_\_TCROSSPROD\_HPP

2 #define STAN\_\_AGRAD\_\_REV\_\_MATRIX\_\_TCROSSPROD\_HPP

3

4 #include <vector>

5 #include <boost/math/tools/promotion.hpp>

6 #include <stan/math/matrix/Eigen.hpp>

7 #include <stan/math/matrix/typedefs.hpp>

8 #include <stan/agrad/rev/var.hpp>

9 #include <stan/agrad/rev/matrix/Eigen\_NumTraits.hpp>

10 #include <stan/agrad/rev/matrix/typedefs.hpp>

11 #include <stan/agrad/rev/matrix/dot\_product.hpp>

12 #include <stan/agrad/rev/matrix/dot\_self.hpp>

13

14 namespace stan {

15  namespace agrad {

16

23  inline matrix\_v

24  tcrossprod(const matrix\_v& M) {

25  if (M.rows() == 0)

26  return matrix\_v(0,0);

27  // if (M.rows() == 1)

28  // return M \* M.transpose();

29

30  // WAS JUST THIS

31  // matrix\_v result(M.rows(),M.rows());

32  // return result.setZero().selfadjointView<Eigen::Upper>().rankUpdate(M);

33

34  matrix\_v MMt(M.rows(),M.rows());

35

36  vari\*\* vs

37  = (vari\*\*)memalloc\_.alloc((M.rows() \* M.cols() ) \* sizeof(vari\*));

38  int pos = 0;

39  for (int m = 0; m < M.rows(); ++m)

40  for (int n = 0; n < M.cols(); ++n)

41  vs[pos++] = M(m,n).vi\_;

42  for (int m = 0; m < M.rows(); ++m)

43  MMt(m,m) = var(new dot\_self\_vari(vs + m \* M.cols(),M.cols()));

44  for (int m = 0; m < M.rows(); ++m) {

45  for (int n = 0; n < m; ++n) {

46  MMt(m,n) = var(new dot\_product\_vari<var,var>(vs + m \* M.cols(),

47  vs + n \* M.cols(),

48  M.cols()));

49  MMt(n,m) = MMt(m,n);

50  }

51  }

52  return MMt;

53  }

54

55  }

56 }

57 #endif

typedefs.hpp

typedefs.hpp

Eigen\_NumTraits.hpp

stan::agrad::memalloc\_

memory::stack\_alloc memalloc\_

**Definition:** var\_stack.cpp:16

dot\_self.hpp

dot\_product.hpp

stan::agrad::matrix\_v

Eigen::Matrix< var, Eigen::Dynamic, Eigen::Dynamic > matrix\_v

The type of a matrix holding stan::agrad::var values.

**Definition:** typedefs.hpp:21

stan::agrad::vari

The variable implementation base class.

**Definition:** vari.hpp:28

var.hpp

Eigen.hpp

stan::memory::stack\_alloc::alloc

void \* alloc(size\_t len)

Return a newly allocated block of memory of the appropriate size managed by the stack allocator...

**Definition:** stack\_alloc.hpp:168

stan::agrad::var

Independent (input) and dependent (output) variables for gradients.

**Definition:** var.hpp:27

stan::agrad::tcrossprod

Eigen::Matrix< fvar< T >, R, R > tcrossprod(const Eigen::Matrix< fvar< T >, R, C > &m)

**Definition:** tcrossprod.hpp:17


---

[ Stan Home Page ]

*© 2011–2014,
Stan Development Team.*
